# Maternal Dietary Patterns during Third Trimester in Association with Birthweight Characteristics and Early Infant Growth

**DOI:** 10.1155/2013/786409

**Published:** 2013-12-31

**Authors:** Anna K. Poon, Edwina Yeung, Nansi Boghossian, Paul S. Albert, Cuilin Zhang

**Affiliations:** ^1^Epidemiology Branch, Division of Intramural Population Health Research, Eunice Kennedy Shriver National Institute of Child Health and Human Development, 6100 Executive Boulevard, 7B03, Bethesda, MD 20892, USA; ^2^Department of Epidemiology, Johns Hopkins Bloomberg School of Public Health, 2024 E. Monument Street, Baltimore, MD 21205, USA; ^3^Biostatistics and Bioinformatics Branch, Division of Intramural Population Health Research, Eunice Kennedy Shriver National Institute of Child Health and Human Development, 6100 Executive Boulevard, 7B03, Bethesda, MD 20892, USA

## Abstract

Our analysis examined the impact of maternal dietary patterns and lifestyle factors on markers of fetal growth, specifically birthweight and size for gestational age (small- (SGA) or large-for-gestational age (LGA)). The Infant Feeding Practices Study II, a prospective cohort study, surveyed pregnant women during their 3rd trimester, of which a subgroup (*n* = 893) completed a food frequency questionnaire. Maternal dietary patterns were evaluated by diet scores (Alternative Healthy Eating Index for Pregnancy and alternate Mediterranean diet) and by carbohydrate quality (glycemic index and glycemic load). Poisson regression with robust standard errors was used to examine the relative risk of SGA and separately LGA, with dietary patterns and other lifestyle factors. Linear regression was used to determine the association of birthweight and early infant growth with better dietary patterns. Relative risk of SGA and LGA was not associated with dietary patterns. Birthweight and infant growth were not associated with maternal diet. Smoking, however, increased the risk of delivering an SGA infant (RR = 2.92, 95% CI: 1.58–5.39), while higher prepregnancy BMI increased the risk of delivering an LGA infant (RR = 1.06, 95% CI: 1.03–1.09). Future studies are needed to evaluate whether deficiencies in more specific maternal dietary nutrients play a role in fetal growth.

## 1. Introduction

Fetal growth is an important determinant not only of infant survival but also of future chronic disease risk. Both low and high birthweight have been associated with increased infant mortality and long-term morbidity [1, 2]. Low birthweight has additionally been associated with elevated risk of type 2 diabetes [3], while high birthweight for gestational age has been associated with increased risk of overweight and obesity in adulthood [4, 5]. Due to the lifelong implications of fetal growth defined by size-at-birth, further research is needed to understand its determinants.

Maternal nutrition is the major fuel for fetal growth [6]. While many studies have examined the role of individual nutrients during pregnancy [7–9], recent focus on nutritional epidemiology has shifted from examining the effect of single nutrients to assessing overall diet quality. Assessing nutrition as a dietary index may be more informative as it accounts for the combined effect of nutrients in foods [10]. In this regard, existing analyses on prenatal dietary patterns with birthweight have been scant and findings have been inconsistent [11]. As a result, further research is needed to understand the role of maternal dietary patterns in association with birthweight.

Our objective was to determine the association of overall maternal dietary patterns, as evaluated by the Alternative Healthy Eating Index for Pregnancy (AHEI-P) and the alternate Mediterranean diet (aMED), with birthweight, birthweight-for-gestational age (large and small), and early infant growth by 4–6 months of life in the Infant Feeding Practices Study II (IFPSII). Furthermore, the association of carbohydrate quality and quantity, as measured by the average glycemic index (GI) of diet and glycemic load (GL), was investigated as well.

## 2. Materials and Methods

### 2.1. Study Population

IFPSII (2005–2007) is a longitudinal cohort study, sampling US women from a nationally distributed consumer opinion panel during their 3rd trimester of pregnancy [12]. After delivery, mothers and infants were eligible to be in the study if the infant was a healthy singleton delivered after at least 35 weeks of gestation, weighed at least 5 pounds, and did not stay in the intensive care unit for more than 3 days. Also, neither the mother nor the infant could have a medical condition that affected infant feeding. Longitudinal data were collected through mailed questionnaires from late pregnancy to 12 months postpartum. A subsample of 1,502 women completed and returned a food frequency questionnaire (FFQ) during the 3rd trimester of pregnancy. Of these, 1,032 remained in the study after the exclusions for the above criteria and other disqualifications [12]. Exclusions were also made for caloric intake in the top 2% or bottom 1% of energy intake (corresponding to women with caloric intake above 4,539 kcal and below 606 kcal). The IFPSII study was approved by the FDA institutional Review Board.

### 2.2. Maternal Characteristics

Maternal demographics were either available via the panel database or collected through a short demographic questionnaire [12]. Examined demographic characteristics included: maternal age (years), maternal race (white or nonwhite), education (high school or less, some college, associate or bachelor, or master or more), and poverty index ratio (<185%, 185 to 350%, or ≥350%). The poverty index ratio describes a family's income relative to their poverty threshold; poverty thresholds vary by family size. Index ratios ≤100% indicate that the family is living at or below the poverty level [13]. Prepregnancy weight (kg/m^2^) and smoking (yes or no) during pregnancy were reported in the prenatal questionnaire during the 3rd trimester. Maternal alcohol use (g) and gestational weight gain (kg) were reported in the prenatal FFQ and neonatal questionnaire, respectively.

### 2.3. Maternal Food Frequency Intake

The Diet History Questionnaire (DHQ) is a validated FFQ originally developed by the National Cancer Institute. The DHQ was modified to reflect dietary intake in the past month rather than the past year and included additional foods and nutrients relevant to pregnant women, such as specific types of fish and dietary supplements [12]. As a result, the modified DHQ mailed to women during their 3rd trimester reflects dietary intake between 28 and 36 weeks of gestation. The DHQ assessments were processed using the NCI's Diet*Calc software (version 1.4.3), which produces nutrient, food category, and glycemic load estimates. The two dietary pattern indices calculated from the DHQ data include the Alternative Healthy Eating Index for Pregnancy (AHEI-P), our primary measure of overall dietary patterns, and the alternate Mediterranean diet (aMED). The average glycemic index (GI) and glycemic load (GL) were also derived from DHQ data as measures of carbohydrate quality.

### 2.4. Alternative Healthy Eating Index for Pregnancy (AHEI-P)

The original Healthy Eating Index (HEI) was developed to measure overall dietary patterns based on the 1995 Food Guide Pyramid and Dietary Guidelines for Americans [14]. Since then, the HEI has been modified periodically to reflect changes in dietary recommendations. Our AHEI-P is based on a 130-point scale with 0–10 points awarded for optimal intake of 13 types of foods and nutrients. The score was adapted from the recently updated AHEI-2010 by Chiuve et al. and an earlier pregnancy AHEI score by Rifas-Shiman et al. [15, 16]. To make the AHEI-2010 suitable for dietary assessment in pregnant women, alcohol was excluded, while calcium, folate, and iron were added to the scoring method [16]. Participants received higher scores for higher intakes of healthier components consisting of vegetables, whole fruit, whole grains, nuts and legumes, long-chain (n-3) fats, polyunsaturated fats, folate, calcium, and iron. Higher intakes of less healthy components including sugar-sweetened beverages, red and processed meat, *trans* fat, and sodium received lower scores. For healthier components, the mother's observed intake was divided by the criterion for maximum points and multiplied by 10. To assign higher points for lower intake of less healthy components, the mother's observed intake was divided by the criterion for maximum points, subtracted from 1, and then multiplied by 10. Sodium intake was divided into eleven groups and scored on a 0–10 point scale. All of the above scores for the individual dietary components were then summed to get the total AHEI-P score for each mother with higher scores indicating better dietary patterns. AHEI-P was categorized into tertiles and also examined as a continuous variable.

### 2.5. Alternate Mediterranean Diet (aMED)

The traditional Mediterranean diet, originally derived from dietary intake observed in the Mediterranean region, distinguishes itself from the AHEI-P based on its different nutrient requirements, such as low intake of saturated fats [17]. To measure adherence to the Mediterranean diet in pregnant women, the score was modified to exclude alcohol intake. Based on an 8-point scale, participants received 1 point if their intake of healthier components exceeded the median intake and if their intake of less healthy components was below the median. The median value for each component was based on the distribution of intake within IFPSII. Healthier components included vegetables, legumes, fruits, nuts, whole grains, fish, and the ratio of monounsaturated to saturated fats, while less healthy components consisted of red and processed meats. The points for each category were summed to get the total aMED for each mother with a higher score indicating better adherence. aMED was categorized into low (0–3), moderate (4-5), and high (6–8) adherence groups and also examined as a continuous variable.

### 2.6. Glycemic Index (GI) and Glycemic Load (GL)

GI is a measure of the glycemic effect of a particular food relative to a standard amount of glucose. GL is a measure of both the quality and quantity of carbohydrate consumption and is calculated based on a food's GI [18]. Both GI and GL differ from the AHEI-P and aMED because they solely capture responses to carbohydrates in diet. GL was estimated from each participant's FFQ response using the Diet*Calc software. Overall average GI of a participant's diet was calculated by dividing GL by total carbohydrate intake and multiplying it by 100 [18]. Both GI and GL were categorized into tertiles and examined as continuous variables in analysis.

### 2.7. Size-at-Birth and Infant Growth

Infant's birthweight was reported on the birth screener after delivery. Gestational age was determined based on the mother's expected date of delivery and the infant's birth date, as reported on the birth screener. Infant's birth length and gender were reported on the neonatal questionnaire. Data on gender, birthweight, and gestational age were used to identify infants as small-for-gestational age (SGA, defined as gender-specific birthweight-for-gestational age ≤10th percentile) or large-for-gestational age (LGA, defined as gender-specific birthweight-for-gestational age ≥90th percentile) [19]. Birthweight z-scores and weight-for-length (WFL) z-scores at birth were additionally calculated based on 2000 CDC reference growth charts.

At the 3rd-, 5th-, 7th-, and 12th-month postpartum mailed surveys, mothers reported their infant's weight and length and the date of each measurement based on their last pediatrician's visit. From these questionnaires, weight and length measurements were used to calculate WFL z-scores when the infant's age was between 4 and 6 months using the 2000 CDC growth charts. This age was chosen because change in WFL in the first 6 months of life is a marker for accelerated infant growth.

### 2.8. Statistical Analysis

The final analytic sample included 893 participants after exclusions for gestational ages above 43.5 weeks (*n* = 6), type I or type II diabetes (*n* = 9), or missing gender information (*n* = 66). Associations between tertiles of AHEI-P and maternal covariates were assessed using ANOVA for continuous variables and *χ* ^2^-test for categorical variables. Poisson regression with robust standard errors [20] was used to estimate the relative risks and 95% confidence intervals for the associations of SGA and LGA by AHEI-P tertiles, adjusting for total energy intake, maternal age, race, education, poverty index ratio, maternal prepregnancy BMI, and smoking and alcohol consumption during pregnancy. This analysis was repeated with the aMED categories, GI tertiles, and GL tertiles.

Multiple linear regression analysis was used to determine the association of birthweight, WFL z-score at birth, and WFL z-score at 4–6 months with AHEI-P, aMED, GI, and GL. Models for birthweight and birth WFL z-score were fully adjusted for the above-mentioned covariates with additional adjustment for gestational age. Change in infant growth as an outcome was modeled with WFL z-score at 4–6 months, adjusting for gestational age, WFL z-score at birth, and all the other covariates [16]. All associations are presented with their respective estimates and 95% confidence intervals with statistical significance at *P* < 0.05. Data analyses were performed using SAS 9.3 (SAS Institute Inc., Cary, NC, USA).

## 3. Results

### 3.1. Baseline Characteristics

The mean (±standard deviation) AHEI-P, aMED, GI, and GL values were 59.1 ± 11.8 points, 4.0 ± 1.8 points, 50.0 ± 3.6 percent, and 141.5 ± 66.0 g, respectively. Total food energy increased with higher AHEI-P tertiles (*P* < 0.0001). Women with higher AHEI-P scores tended to be older, with higher education, lower poverty index ratio, and lower prepregnancy BMI (Tables 1 and 2). Women excluded from the study sample did not have significantly different mean scores of prenatal dietary indices from those included in the study (data not shown).

### 3.2. Association of AHEI-P with Size-for-Gestational Age

Eighty-two (9.2%) infants were LGA. No significant associations between AHEI-P tertiles and LGA were observed. The relative risk of the highest versus the lowest tertile of AHEI-P was 0.92 (95% confidence interval (CI): 0.50–1.69). LGA, however, was associated with prepregnancy BMI (Relative Risk (RR) = 1.06, 95% CI: 1.03–1.09). aMED, GI, and GL were not associated with LGA (Table 3).

Seventy-one (8.0%) infants were SGA. Similarly, after adjusting for covariates, no significant differences were examined across the tertiles of AHEI-P in association with SGA risk. The risk estimate of the highest tertile compared to the lowest tertile of AHEI-P was 0.93 (95% CI: 0.49–1.75). SGA, however, was associated with maternal race (RR = 3.16, 95% CI: 1.97–5.06) and smoking status (RR = 2.92, 95% CI: 1.58–5.39). aMED, GI, and GL were not associated with SGA (Table 3). Stratifying by smoking status did not alter our findings with AHEI-P and neither did excluding women with gestational diabetes (*n* = 46) (data not shown).

### 3.3. Association of AHEI-P with Birthweight, Birth WFL, and Change in WFL z-Scores

Neither birthweight z-scores nor birth WFL z-scores were associated with AHEI-P scores (Table 4). The mean differences in z-score units per unit increase in AHEI-P were 0.002 (95% CI: −0.003–0.008) and 0.005 (95% CI: −0.004–0.013), respectively. AHEI-P was also not associated with change in WFL at 4–6 months (difference = 0.009, 95% CI: −0.004–0.023). Fully adjusted models with aMED, GI, and GL were similarly not associated with birthweight, birth WFL, and change in WFL z-scores at 4–6 months.

## 4. Discussion

In a nationally distributed sample of 893 US mothers of healthy singletons delivered after 35 weeks of gestation, maternal dietary patterns and carbohydrate quality during third trimester were not associated with offspring outcomes consisting of birthweight, size-for-gestational age (small or large), and infant growth in the first 4–6 months of life. On the other hand, maternal characteristics including race, smoking status, and prepregnancy BMI were associated with SGA and LGA risk.

Two previous studies have examined dietary patterns as measured by AHEI-P in association with birthweight outcomes. In the Infancia y Medio Ambiente (INMA) cohort of 787 pregnant Spanish women, investigators found a reduced risk of fetal growth restriction (FGR) for weight (defined as infants below the lower limit of the 80% CI for predicted birthweight based on maternal and paternal anthropometry) per five-point increase in AHEI-P (Odds Ratio (OR) = 0.70, 95% CI: 0.59–0.85) [21]. Better dietary patterns also predicted higher birthweight (*P* trend = 0.009) [21]. However, findings from the US, including the results of our analysis indicating no association, have not been as supportive of such an effect. In Project Viva, a prospective cohort of 1,777 pregnant US women, a nonsignificant lowering of risk of both SGA (OR = 0.92, 95% CI: 0.82–1.02) and LGA (OR = 0.95, 95% CI: 0.89–1.02) was identified per five-point increase in AHEI-P [16]. For comparison, reproducing the analysis in IFPSII with logistic regression and AHEI-P (per 5-point increment) did not alter the nonsignificant association with either SGA (OR = 0.98, 95% CI: 0.86–1.12) or LGA (OR = 0.99, 95% CI: 0.88–1.12). As for measuring dietary patterns by the aMED, the INMA-Mediterranean cohort found lower risk (RR = 0.50, 95% CI: 0.28–0.90) of delivering an infant with FGR for weight (defined as infants below the 10th percentile of the predicted birthweight distribution). The results, however, were not consistently protective in the INMA-Atlantic (RR = 0.97, 95% CI: 0.42–2.26) or RHEA cohorts (RR = 1.96, 95% CI: 0.90–4.25) [22]. Also, a recent systematic review of eight studies examining low GI and GL diets suggests that further research is needed before recommending either pattern during pregnancy [23]. These findings taken together with our current analysis from IFPSII suggest that there remains a lack of evidence that prenatal dietary patterns during third trimester, as measured by the AHEI-P and aMED, and carbohydrate quality, based on GI and GL, can prevent SGA or LGA.

Our observation that prepregnancy weight affects birthweight suggests that diet is not completely irrelevant. As prepregnancy BMI is associated with infant size-at-birth, this association suggests prepregnancy diet may be related to fetal growth. In our study, mothers had a 6% higher risk (95% CI: 1.03–1.09) of delivering an LGA infant per unit increase in BMI. These findings are consistent with previous studies [24–28]. Djelantik et al., for example, reported that overweight (RR = 1.55, 95% CI: 1.30–1.84) and obese (RR = 2.03, 95% CI: 1.60–2.59) women were significantly more likely to deliver an LGA infant compared to women with normal prepregnancy BMI [26]. Other authors additionally reported a positive association of underweight prepregnancy BMI (BMI < 18.5 kg/m^2^) with intrauterine growth restriction compared to women with normal prepregnancy BMI (18.5 ≤ BMI < 25) [29]. These findings thus emphasize the importance of maternal prepregnancy weight as an early indicator of identifying women who may be at risk of delivering infants with abnormal birthweight.

Among pregnant US women with sufficient energy intake, smoking during pregnancy increased SGA risk. This result indicates that growth *in utero* depends on maternal factors other than prenatal dietary patterns. Smoking is a well-known modifiable risk factor for restricting fetal growth and our findings for smoking are in agreement with previous studies [30, 31]. Our findings show that mothers who reported smoking during pregnancy were about three times (95% CI: 1.58–5.39) more likely to deliver an SGA infant. Similarly, another study reported that mothers who smoked during pregnancy delivered infants who were 142 g lighter on average and two times (RR = 2.07, 95% CI: 1.69–2.53) more likely to be growth restricted than nonsmoking mothers [31]. These results are evidence of the adverse effects of maternal smoking habits on fetal growth and add to the mounting evidence of the importance of smoking cessation.

Our analysis should be interpreted in the context of the strengths and limitations of the study. Two key strengths include the prospective study design with the assessment of prenatal diet prior to the examined birth outcomes and the evaluation of maternal diet using validated measures of dietary patterns. There were also several limitations. First, while prenatal diet was only measured once during the third trimester, repeated measurements may have better captured dietary intake. Dietary intake in both the INMA and Project Viva cohorts reflected intake in the first trimester. However, we do not expect maternal diet to differ significantly between early and late pregnancy [32]. As such, differences in our findings in comparison to these cohorts are unlikely to be due to the exposure window. Second, gestational age was self-reported. Third, our sample size may have limited the statistical power of detecting a significant association. However, our sample size (*n* = 893) was comparable to the INMA cohort (*n* = 787). Fourth, the IFPSII population largely consisted of healthy singletons, which restricts the generalizability of our results.

Our findings suggest that prenatal dietary patterns as measured by AHEI-P and aMED, and carbohydrate quality based on GI and GL, during the 3rd trimester do not affect birth outcomes defined by birthweight, SGA and LGA, as well as infant growth at 4–6 months. Our results continue to emphasize the importance of prepregnancy BMI and smoking habits as key risk factors for SGA and LGA and as such continue to be clinically useful indicators for identifying women at risk for poor birth outcomes.

## Figures and Tables

**Table 1:** Alternative Healthy Eating Index for Pregnancy (AHEI-P)^a^ scoring method and mean AHEI-P scores for 893 women in the Infant Feeding Practices Study II.

Component	Criterion for minimum (0)	Criterion for maximum (10)	Mean score ± SD
Vegetables, servings/d	0	≥5	5.0 ± 2.7
Whole fruit, servings/d	0	≥4	4.4 ± 3.1
Whole grains, g/d	0	75	2.5 ± 1.5
Sugar-sweetened beverages, servings/d	≥1	0	2.0 ± 3.3
Nuts and legumes, servings/d	0	≥1	3.7 ± 2.8
Red/processed meat, servings/d	≥1.5	0	1.4 ± 2.3
*trans* Fat, % of energy	≥4	≤0.5	5.5 ± 1.6
Long-chain (*n* = 3) fats (EPA + DHA), mg/d	0	250	2.5 ± 2.2
PUFA, % of energy	≤2	≥10	5.6 ± 2.1
Sodium, mg/d	Highest decile	Lowest decile	5.2 ± 3.1
Calcium, mg/d	0	≥1200	8.3 ± 2.2
Folate, mcg/d	0	≥600	6.9 ± 2.2
Iron, mg/d	0	≥27	6.0 ± 2.1
AHEI-P	—	—	59.1 ± 11.8

^a^The AHEI-P score was adapted from Rifas-Shiman et al. 2009 and Chiuve et al. 2012 [15, 16]; out of 130 points.

**Table 2:** Baseline characteristics of maternal covariates by tertiles of AHEI-P.

	T1	T2	T3	*P*-value	Overall
*Maternal demographics *					
Age, years	28.4 ± 5.3	28.7 ± 5.1	30.1 ± 5.5	<0.001	29.1 ± 5.4
Race, *n* (%)					
White	264 (89.5)	261 (87.9)	251 (84.8)	0.22	777 (87.4)
Education, *n* (%)					
High school or less	68 (23.9)	42 (14.7)	43 (15.3)	<0.001	154 (18.0)
Some College	126 (44.2)	108 (37.8)	102 (36.2)		336 (39.3)
Associate or BA	73 (25.6)	97 (33.9)	101 (35.8)		271 (31.7)
Master or more	18 (6.3)	39 (13.6)	36 (12.8)		93 (10.9)
Poverty index ratio, *n* (%)					
<185%	138 (46.5)	105 (35.2)	92 (31.0)	<0.0001	335 (37.5)
185 to 350%	114 (38.4)	118 (39.6)	113 (38.1)		346 (38.8)
≥350%	45 (15.2)	75 (25.2)	92 (31.0)		212 (23.7)
*Maternal health *					
Smoked, *n* (%)	31 (10.5)	25 (8.5)	17 (5.8)	0.11	74 (8.3)
Alcohol, g	0.1 ± 0.3	0.1 ± 0.2	0.2 ± 1.5	0.16	0.1 ± 0.9
Prepregnancy BMI, kg/m^2^	27.5 ± 7.3	25.5 ± 5.8	25.3 ± 5.7	<0.0001	26.1 ± 6.4
Gestational weight gain, kg	13.4 ± 6.4	14 ± 6.1	14.4 ± 5.8	0.12	14.0 ± 6.1
Total food energy, kcal	1733 ± 660	2036 ± 729	2546 ± 969	<0.0001	2104 ± 864
*Infant Health *					
Gestational age, weeks	39.3 ± 1.2	39.3 ± 1.3	39.3 ± 1.2	0.62	39.3 ± 1.2
Birthweight					
Birthweight, g	3444 ± 446	3405 ± 462	3478 ± 454	0.14	3443 ± 454
BW ≤10th percentile, *n* (%)	24 (8.1)	23 (7.7)	24 (8.1)	0.98	71 (7.95)
BW ≥90th percentile, *n* (%)	32 (10.8)	22 (7.4)	28 (9.4)	0.35	82 (9.18)

Diet scores					
AHEI-P, range (*n*)	33–52 (297)	53–62 (298)	63–98 (297)		59.1 ± 11.8
aMED^a^, range (*n*)	0–3 (369)	4-5 (333)	6–8 (191)		4.0 ± 1.8

Carbohydrate quality					
GI^b^, range (*n*)	35–48 (297)	49–51 (298)	52–63 (298)		50.0 ± 3.6
GL^c^, range (*n*)	38–107 (297)	108–152 (298)	153–520 (298)		141.5 ± 66.0

^a^Alternate Mediterranean diet, out of 8 pts; ^b^glycemic index; ^c^glycemic load.

**Table 3:** Risk ratios and 95% confidence intervals for the association of SGA and LGA with dietary pattern indices.

	SGA (*n* = 71)	LGA (*n* = 82)
	Estimate (95% CI)	Estimate (95% CI)
*Model with AHEI-P* ^ a^		
*n* (number of obs. used)	755	775
AHEI-P		
T2 (53–62) versus T1 (33–52)	0.73 (0.41, 1.31)	0.74 (0.43, 1.26)
T3 (63–98) versus T1 (33–52)	0.93 (0.49, 1.75)	0.92 (0.50, 1.69)
Maternal age	0.95 (0.90, 1.01)	0.99 (0.96, 1.03)
Nonwhite versus White	**3.16 (1.97, 5.06)**	0.43 (0.16, 1.15)
Education		
Some college versus ≤HS	1.29 (0.68, 2.43)	1.15 (0.61, 2.20)
Associate or BA versus ≤HS	0.71 (0.30, 1.68)	1.62 (0.81, 3.23)
Master or more versus ≤HS	1.03 (0.35, 3.08)	1.39 (0.57, 3.37)
Poverty index ratio		
(185 to 350%) versus (<185%)	1.16 (0.63, 2.12)	1.18 (0.70, 1.97)
(≥350%) versus (<185%)	1.83 (0.91, 3.65)	0.89 (0.45, 1.73)
Smoked, yes versus no	**2.92 (1.58, 5.39)**	0.76 (0.29, 1.97)
Alcohol, g	0.97 (0.82, 1.15)	0.47 (0.10, 2.13)
Total energy	1.00 (1.00, 1.00)	1.00 (1.00, 1.00)
Prepregnancy BMI, kg/m^2^	0.98 (0.94, 1.02)	**1.06 (1.03, 1.09)**
*Model with aMED* ^ a^		
T2 (4-5) versus T1 (0–3)	0.75 (0.44, 1.29)	0.71 (0.44, 1.14)
T3 (6–8) versus T1 (0–3)	0.94 (0.48, 1.81)	0.71 (0.37, 1.35)
*Model with GI* ^ a^		
T1 (35–48) versus T3 (52–63)	0.86 (0.49, 1.49)	1.03 (0.60, 1.75)
T2 (49–51) versus T3 (52–63)	0.87 (0.50, 1.50)	1.26 (0.77, 2.08)
*Model with GL* ^ a^		
T1 (38–107) versus T3 (153–520)	0.92 (0.35, 2.40)	1.18 (0.50, 2.78)
T2 (108–152) versus T3 (153–520)	1.13 (0.53, 2.37)	1.10 (0.56, 2.17)

^a^Adjusted for total energy intake, race, education, age, poverty index ratio, smoking, alcohol, and pre-pregnancy BMI.

**Table 4:** Mean differences in birth weight, weight-for-length (WFL), and change in WFL z-scores per unit increase in dietary pattern indices.

	Birthweight^a^	Birth WFL^a^	Change in WFL at 4–6 months^b^
	Estimate (95% CI)	Estimate (95% CI)	Estimate (95% CI)
*n* (number of obs. used)	815	815	426
AHEI-P	0.002 (−0.003, 0.008)	0.005 (−0.004, 0.013)	0.009 (−0.004, 0.023)
aMED	−0.003 (−0.036, 0.031)	0.03 (−0.03, 0.08)	0.06 (−0.03, 0.14)
GI	−0.015 (−0.031, 0.001)	−0.02 (−0.04, 0.01)	−0.01 (−0.04, 0.03)
GL	−0.002 (−0.004, 0.001)	0.0004 (−0.0028, 0.0036)	−0.003 (−0.009, 0.002)

^a^Adjusted for total energy intake, race, education, age, poverty index ratio, smoking, alcohol, pre-pregnancy BMI, and gestational age; ^b^also adjusted for birth WFL.
